# Comparative Effectiveness of Abiraterone and Enzalutamide in Patients With Metastatic Castration-Resistant Prostate Cancer in Taiwan

**DOI:** 10.3389/fonc.2022.822375

**Published:** 2022-03-07

**Authors:** Pei-Yu Li, Ying-Hao Lu, Chung-Yu Chen

**Affiliations:** ^1^ Master Program in Clinical Pharmacy, School of Pharmacy, Kaohsiung Medical University, Kaohsiung, Taiwan; ^2^ Department of Pharmacy, Kaohsiung Medical University Hospital, Kaohsiung, Taiwan; ^3^ Department of Medical Research, Kaohsiung Medical University Hospital, Kaohsiung, Taiwan; ^4^ Center for Big Data Research, Kaohsiung Medical University, Kaohsiung, Taiwan

**Keywords:** castration-resistant prostate cancer, hormone therapy, abiraterone, enzalutamide, real-world data, comparative effectiveness

## Abstract

**Background:**

Abiraterone and enzalutamide are widely used as first-line treatment for metastatic castration-resistant prostate cancer (mCRPC); however, their efficacy in mCRPC has been inconsistently demonstrated in other outcome studies from real-world databases. The aim of our study was to assess the comparative effectiveness of abiraterone and enzalutamide in patients with mCRPC using real-world data from Taiwan.

**Methods:**

This retrospective cohort population-based study included patients identified in the Taiwan National Health Insurance Research Database who had been diagnosed with mCRPC and who had taken abiraterone or enzalutamide between December 2014 and August 2017. The study’s outcome evaluated the differences in overall survival (OS) and time to treatment failure (TTF) between abiraterone and enzalutamide over a 15-month follow-up period. The patients were followed from the index date to when the outcome occurred, to December 31, 2018, or to the patients’ withdrawal from the National Health Insurance program. The estimated relative treatment effects of abiraterone and enzalutamide on OS and TTF were adjusted by the inverse probability of treatment weighting (IPTW) using the Kaplan–Meier method and a Cox proportional hazards model.

**Results:**

The abiraterone and enzalutamide groups consisted of 1,046 and 118 patients, respectively. After IPTW adjustment, 1,164 patients in the abiraterone group and 1,158 in the enzalutamide group underwent an outcome evaluation. Enzalutamide showed a similar OS rate to that of abiraterone (57.58% vs. 49.51%, *p* = 0.095 by log-rank test). Enzalutamide significantly reduced the risk of death for mCRPC when compared with abiraterone [adjusted hazard ratio (aHR), 0.828; 95% CI 0.731–0.938]. However, similar results were not observed in the TTF outcomes (63.84% vs. 67.79%, *p* = 0.2651 by log-rank test; aHR, 0.902; 95% CI 0.812–1.002).

**Conclusion:**

In conclusion, enzalutamide was associated with better OS for mCRPC than abiraterone in the Taiwan population. Our study showed that there was no statistically significant difference in TTF between enzalutamide and abiraterone. Studies with longer surveillance of enzalutamide and abiraterone using real-world databases are needed.

## Introduction

Prostate cancer is the second most common cancer in men worldwide (1). In Taiwan, the cancer registry data indicated that prostate cancer ranked fifth in terms of incidence among cancers for men in 2018, with a mortality rate of 11.49 per 100,000 inhabitants (2). Generally, androgen deprivation therapy (ADT) is the first-line treatment for men with advanced prostate cancer. However, most patients eventually progress to castration-resistant prostate cancer (CRPC). According to the results of a systematic review, 10%–20% of patients with prostate cancer progressed to CRPC within 5 years. Metastatic CRPC (mCRPC) accounts for approximately 84% of such cases (3). Before 2001, most medical treatments for mCRPC were either the continuous use of ADT or docetaxel as chemotherapy. Recently, several international clinical trials have established the therapeutic efficacy of second-generation hormone therapy in prostate cancer, including abiraterone as an androgen synthesis inhibitor and enzalutamide as an androgen receptor (AR) antagonist. The favorable clinical activity of abiraterone and enzalutamide was established in phase III trials in men with mCRPC, and both drugs have been shown to improve OS in men who had been previously treated with docetaxel (4, 5) and in those who were chemotherapy naive (6, 7). Based on the results of these clinical trials, abiraterone and enzalutamide have become the preferred approach and are widely used as first-line treatment for mCRPC. In Taiwan, abiraterone and enzalutamide were introduced in 2012 and 2015, respectively, for treating mCRPC.

To date, no clinical trial has performed a direct treatment efficacy comparison between abiraterone and enzalutamide; however, there have been a number of studies indirectly comparing the treatments for mCRPC (8). Several retrospective studies have also compared the effectiveness of abiraterone and enzalutamide in real-world settings; however, their efficacy in mCRPC has been inconsistently demonstrated in other outcome studies from real-world databases (9, 10). A number of the studies showed that the two drugs were comparable in survival outcomes (11–13), while others indicated that enzalutamide was the preferable option (8, 14). In addition to the inconsistent results, several studies have indicated cross-resistance between abiraterone and enzalutamide (15, 16). Taiwan’s National Health Insurance (NHI) requires that these two drugs must not be interchanged. Consequently, determining which of the drugs should be considered the first choice when treating mCRPC is important. However, information on the effectiveness of the two drugs in Taiwanese patients is still inadequate. The present study therefore employed the NHI Research Database (NHIRD) to evaluate the comparative effectiveness of abiraterone and enzalutamide in patients with mCRPC in Taiwan.

## Materials and Methods

### Data Source

This is a population-based retrospective cohort study that analyzed data from the NHIRD provided by the Ministry of Health and Welfare in Taiwan. The NHIRD contains encrypted data on outpatient care claims, hospital inpatient care, ambulatory care, dental services, and prescription drug records. Taiwan’s NHI program was launched as a single-payer system on March 1, 1995, and has enrolled over 99.9% of Taiwan’s population. The study used the medical records of the full population database from January 2011 to December 2018. The study was conducted according to the Declaration of Helsinki and was approved by the Institutional Review Board of Kaohsiung Medical University Hospital (Research Ethics Committee No. KMUHIRB-E(II)-20210108), which waived the requirement for written informed consent.

### Patients

We identified patients aged older than 20 years who had an inpatient or outpatient first diagnosis of prostate cancer [International Classification of Diseases - Clinical Modification (ICD-9-CM) code 185 or ICD-10-CM C61] between 2014 and 2017. The population with prostate cancer who were initially prescribed abiraterone or enzalutamide between December 1, 2014, and August 31, 2017, were identified. The index date was defined as the first date one of the two study drugs was prescribed. Since the regulation of the NHI payment guideline in Taiwan, abiraterone and enzalutamide could only be indicated for patients with mCRPC with an Eastern Cooperative Oncology Group score ≤2. The NHI policy also required that the administration of abiraterone or enzalutamide be approved through pre-examination, which had to be reviewed every 3 months. When applying for the pre-examination, several documents need to be attached. Taiwan’s insurance program restricts the use of abiraterone and enzalutamide, a situation conducive to verifying the study’s target population. We excluded patients with some other cancer diagnosed in the year prior to the index date or who had been prescribed both abiraterone and enzalutamide at the index date. Patients with CRPC in this study cohort were divided into two treatment groups according to their first prescription of abiraterone or enzalutamide.

### Outcomes Measured

The effectiveness outcomes were overall survival (OS) and time to treatment failure (TTF). The follow-up period for OS was estimated from the index date to the date of death or censoring, whichever occurred first. Censoring was defined as the end date of follow-up or the date on which the patients switched from abiraterone to enzalutamide (and vice versa) within a 60-day period. The follow-up period for TTF was calculated from the index date to the date of treatment failure or censoring, whichever occurred first. The treatment failure events included the discontinuation of abiraterone or enzalutamide for any reason over the 60-day period, the addition of other treatments for prostate cancer, newly diagnosed metastatic cancer, and prostate cancer-related death. The definition of other treatments for prostate cancer was the addition of chemotherapy (docetaxel or mitoxantrone), ketoconazole, or ADT therapy that included antiandrogens, estrogens, or antiandrogen withdrawal. Patients who switched from abiraterone to enzalutamide (and vice versa) within the 60-day period or who died from other causes were censored. The condition of discontinuation in the NHI payment regulation was defined as not achieving a 50% decrease in prostate-specific antigen (PSA) levels or an increase in PSA levels >50% from the nadir in the following pre-examination, which could be referred to as PSA response and PSA progression (defined by the Prostate Cancer Clinical Trials Working Group), respectively. All eligible patients were followed from the index date to the first occurrence of the event; the end of the 15-month follow-up; December 31, 2018; or their withdrawal from the NHI program, whichever came first.

### Data Collection

We extracted the following study variables from the database: age, age group (45–64, 65–84, ≥85 years), previous treatments (none, radical prostatectomy/radiation therapy only, hormone only, and hormone plus radical prostatectomy/radiation therapy), metastasis sites (no bone/viscera, bone, viscera, bone/viscera), ADT duration, Charlson Comorbidity Index score (≤7 or >7), and comorbidities. The ADT duration was calculated from the first record of orchiectomy or medical castration to the index date. The patients were stratified into two groups according to ADT duration ≤12 and >12 months. Comorbidities were defined as at least two outpatient care visit records or one inpatient diagnosis record in the year prior to the index date. The ICD codes used for the comorbidities are listed in **Supplementary Table S2**.

### Statistical Analysis

The descriptive statistics are presented as mean ± standard deviation and median with interquartile range (IQR) for the continuous variables, while numbers and percentages were employed for the categorical variables. The standardized mean difference (SMD) was used for the categorical and continuous variables to explore the differences in baseline characteristics between the abiraterone and enzalutamide groups.

To decrease the probable selection bias among the abiraterone and enzalutamide therapies, we performed inverse probability of treatment weighting (IPTW) for the OS and TTF estimates (17). The propensity score calculated by logistic regression was created to adjust the baseline differences. The selection of covariates included in the propensity model was based on the potential prognostic baseline factors for the efficacy outcome when reviewing related studies. The covariates included in this model were age group, metastasis site, previous ADT, and number of docetaxel cycles previously undergone. After performing the IPTW method based on the propensity score, all of the results were analyzed with the reweighted population. The weighted approach ensured that the patients would not be excluded and mimicked a pseudo-population to reflect the baseline characteristics of the whole population.

Kaplan–Meier curves with the log-rank test were used to estimate the OS, TTF, and differences between the two groups. We performed a Cox proportional hazards regression to estimate the hazard ratio (HR) and 95% confidence interval (CI) in the univariate and multivariable analyses. The characteristics used for the adjustment included age group, metastasis site, previous treatment for prostate cancer, and docetaxel cycles. We used SAS 9.4 software (SAS Institute Inc., Cary, NC, USA) to perform all of the data analysis for the study. An SMD <0.10 indicated an acceptable baseline balance between the study groups. A two-sided *p*-value <0.05 was considered statistically significant.

### Sensitivity Analysis

Based on the results of clinical trials on abiraterone and enzalutamide, a 15-month follow-up might not be long enough to observe the survival outcome due to the database’s limitations. The follow-up was therefore extended in the sensitivity analysis. The outcome analyses of the 24-month follow-up were also reported in the study.

## Results

### Patient Characteristics

Overall, we identified 1,379 patients diagnosed with prostate cancer and with at least one prescription of abiraterone or enzalutamide from December 1, 2014, to August 31, 2017. All candidates were 20 years of age or older at the index date. After excluding the patients diagnosed with another cancer in the year prior to the index date (*n* = 215) and those prescribed both abiraterone and enzalutamide at the index date (*n* = 0), the study population consisted of 1,164 patients, with 1,046 in the abiraterone group and 118 in the enzalutamide group (**Supplementary Figure S1**).

**Table 1** lists the study population’s baseline characteristics. The mean/median age was 72.77/73 and 72.10/72 years for the abiraterone and enzalutamide groups, respectively, with no statistically significant differences in mean age between the groups; however, there was a statistically significant difference in the distribution of the age groups. There were also significant differences in the distribution of the metastasis site, previous prostate cancer treatment, docetaxel cycles, and ADT duration. The abiraterone group had undergone more docetaxel cycles before the index date and had a significantly longer ADT duration. As for comorbidities, the enzalutamide group had a significantly higher rate of hypertension and dyslipidemia. There were significant intergroup differences in the distribution of diabetes mellitus, liver disease, coronary artery disease, chronic heart failure, chronic kidney disease, and chronic obstructive pulmonary disease. There was also no statistically significant difference in the distribution of the Charlson Comorbidity Index scores. The population’s baseline characteristics after IPTW are shown in **Supplementary Table S1**.

**Table 1:** Baseline characteristics.

	*N*	%	Abiraterone		Enzalutamide		SMD
			*N*	%	*N*	%	
Total	1,164		1,046		118		
Age (years)							
Mean (± SD)	72.71	8.74	72.77	8.79	72.1	8.29	0.08
45–64	234	20.10	216	20.65	18	15.25	0.14
65–84	837	71.91	744	71.13	93	78.81	0.18
≥85	93	7.99	86	8.22	7	5.93	0.09
Metastasis site							
No bone/visceral	504	43.30	456	43.59	48	40.68	0.06
Bone metastasis	512	43.99	458	43.79	54	45.76	0.04
Visceral metastasis	41	3.52	33	3.15	8	6.78	0.17
Bone + visceral	107	9.19	99	9.46	8	6.78	0.10
Previous treatment							
None	5	0.43	5	0.48	0	0	–
RP/RT only	1	0.09	1	0.10	0	0	–
Hormone only	851	73.11	758	72.47	93	78.81	0.15
Hormone + RP/RT	307	26.37	282	26.96	25	21.19	0.14
Docetaxel cycles							
0	26	2.23	20	1.91	6	5.08	0.17
1–7	424	36.43	376	35.95	48	40.68	0.10
≥8	714	61.34	650	62.14	64	54.24	0.16
ADT duration							
≤12 months	77	6.62	66	6.31	11	9.32	0.12
>12 months	1,087	93.38	980	93.69	107	90.62	
CCI score							
≤7	415	35.65	376	35.95	39	33.05	0.06
>7	749	64.35	670	64.05	79	66.95	
Comorbidity							
Hypertension	608	52.23	541	51.72	67	56.78	0.10
Dyslipidemia	239	20.53	200	19.12	39	33.05	0.32
Diabetes mellitus	275	23.63	246	23.52	29	24.58	0.02
Liver disease	98	8.42	89	8.51	9	7.63	0.03
Stroke	62	5.33	62	5.93	0	0	–
Coronary artery disease	172	14.78	154	14.72	18	15.25	0.01
Congestive heart failure	52	4.47	46	4.40	6	5.08	0.03
Chronic kidney disease	106	9.11	97	9.27	9	7.63	0.06
COPD	93	7.99	86	8.22	7	5.93	0.09

SMD, standard mean difference; SD, standard deviation; RP, radical prostatectomy; RT, radiation therapy; ADT, androgen deprivation therapy; CCI, Charlson Comorbidity Index; COPD, chronic obstructive pulmonary disease.

### Overall Survival

**Figure 1:**
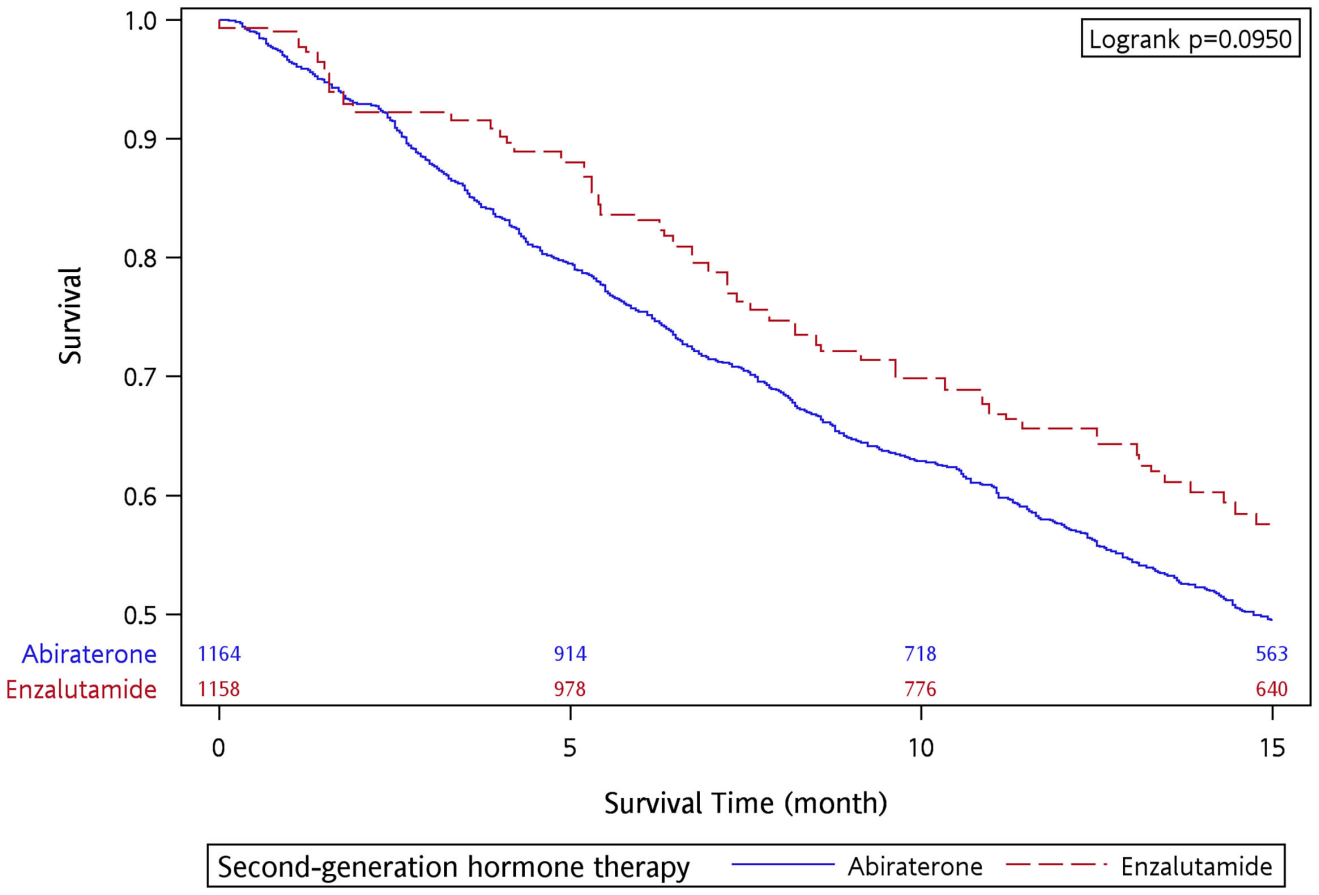
Overall survival of abiraterone and enzalutamide in the 15-month follow-up period after applying IPTW.

There were 694 and 71 deaths within the 15-month follow-up in the abiraterone and enzalutamide groups, respectively. The OS rate at 15 months was 49.58% and 55.88% for the abiraterone and enzalutamide groups, respectively. The median follow-up was 14.43 and 15 months for the abiraterone and enzalutamide groups, respectively. The OS curve is presented in **Supplementary Figure S2**. After applying IPTW, the OS rate at 15 months was 49.51% and 57.58% for the abiraterone and enzalutamide groups, respectively. There was no significant difference in OS with a 15-month follow-up between the two groups (*p* = 0.095 by log-rank test) (**Figure 1**).

**Table 2** shows the results of the univariate and multivariable analyses after performing IPTW. The univariate analysis showed that the enzalutamide group had a lower mortality risk than the abiraterone group (crude HR, 0.769, 95% CI 0.681–0.869, *p* < 0.0001). The results of the multivariable analysis showed that the survival effectiveness of enzalutamide was superior to that of abiraterone (adjusted HR, 0.828, 95% CI 0.731–0.938, *p* = 0.003), which was consistent with the result of the univariate analysis. The results of the multivariate analysis also showed that older age, metastasis site, ADT duration, dyslipidemia, diabetes mellitus, and liver disease were risk factors related to mortality in mCRPC treated with second-generation hormone therapy.

**Table 2:** Cox proportional hazards model for overall survival after IPTW.

	*N*	Number of deaths (%)	Univariate analysis			Multivariate analysis		
			Crude HR	95% CI	*p*-value	Adjusted HR	95% CI	*p*-value
Group								
Abiraterone	1,164	580 (49.83)						
Enzalutamide	1,158	459 (39.64)	0.769	0.681–0.869	<0.0001	0.828	0.731–0.938	0.003
Age (years)								
45–64	467	199 (42.61)						
65–84	1,655	746 (45.08)	1.064	0.902–1.255	0.4652	1.012	0.856–1.196	0.8868
≥85	199	108 (54.16)	1.355	1.071–1.714	0.0112	1.422	1.111–1.819	0.0052
Metastasis site								
No bone/visceral	1,027	363 (35.32)						
Bone metastasis	1,018	515 (50.61)	1.680	1.469–1.922	<0.0001	1.345	1.050–1.723	0.0188
Visceral metastasis	83	49 (59.88)	2.306	1.713–3.105	<0.0001	2.051	1.429–2.944	<0.0001
Bone + visceral	194	126 (64.79)	2.988	2.438–3.663	<0.0001	2.178	1.618–2.931	<0.0001
Previous ADT								
No	6	4 (66.67)						
Yes	2,316	1,049 (45.29)	0.564	0.211–1.504	0.2523	1.168	0.425–3.21	0.7631
Docetaxel cycles								
0	53	36 (66.74)						
1–7	849	409 (48.13)	0.544	0.387–0.767	0.0005	0.883	0.607–1.285	0.5161
≥8	1,420	609 (42.9)	0.430	0.307–0.602	<0.0001	0.719	0.496–1.041	0.0807
ADT duration								
≤12 months	157	117 (74.85)						
>12 months	2,165	1,338 (61.77)	0.456	0.372–0.56	<0.0001	0.521	0.414–0.655	<0.0001
CCI score								
≤7	850	485 (57.01)						
>7	1,472	970 (65.91)	1.835	1.603–2.101	<0.0001	1.259	0.973–1.629	0.0794
Comorbidity								
Hypertension	1,221	541 (44.34)	0.951	0.842–1.073	0.4119			
Dyslipidemia	595	230 (38.71)	0.768	0.663–0.888	0.0004	0.720	0.617–0.841	<0.0001
Diabetes mellitus	545	281 (51.51)	1.237	1.079–1.418	0.0023	1.337	1.155–1.548	<0.0001
Liver disease	199	103 (51.64)	1.324	1.081–1.623	0.0068	1.249	1.016–1.535	0.0346
Stroke	69	35 (49.78)	1.103	0.786–1.549	0.5705			
Coronary artery disease	328	139 (42.44)	0.908	0.76–1.085	0.2887			
Congestive heart failure	100	42 (42.66)	0.928	0.683–1.262	0.6352			
Chronic kidney disease	193	105 (54.03)	1.369	1.119–1.676	0.0023	1.173	0.949–1.450	0.1394
COPD	165	68 (41.52)	0.912	0.713–1.165	0.4589			

HR, hazard ratio; CI, confidence interval; ADT, androgen deprivation therapy; CCI, Charlson Comorbidity Index.

### Time to Treatment Failure

**Figure 2:**
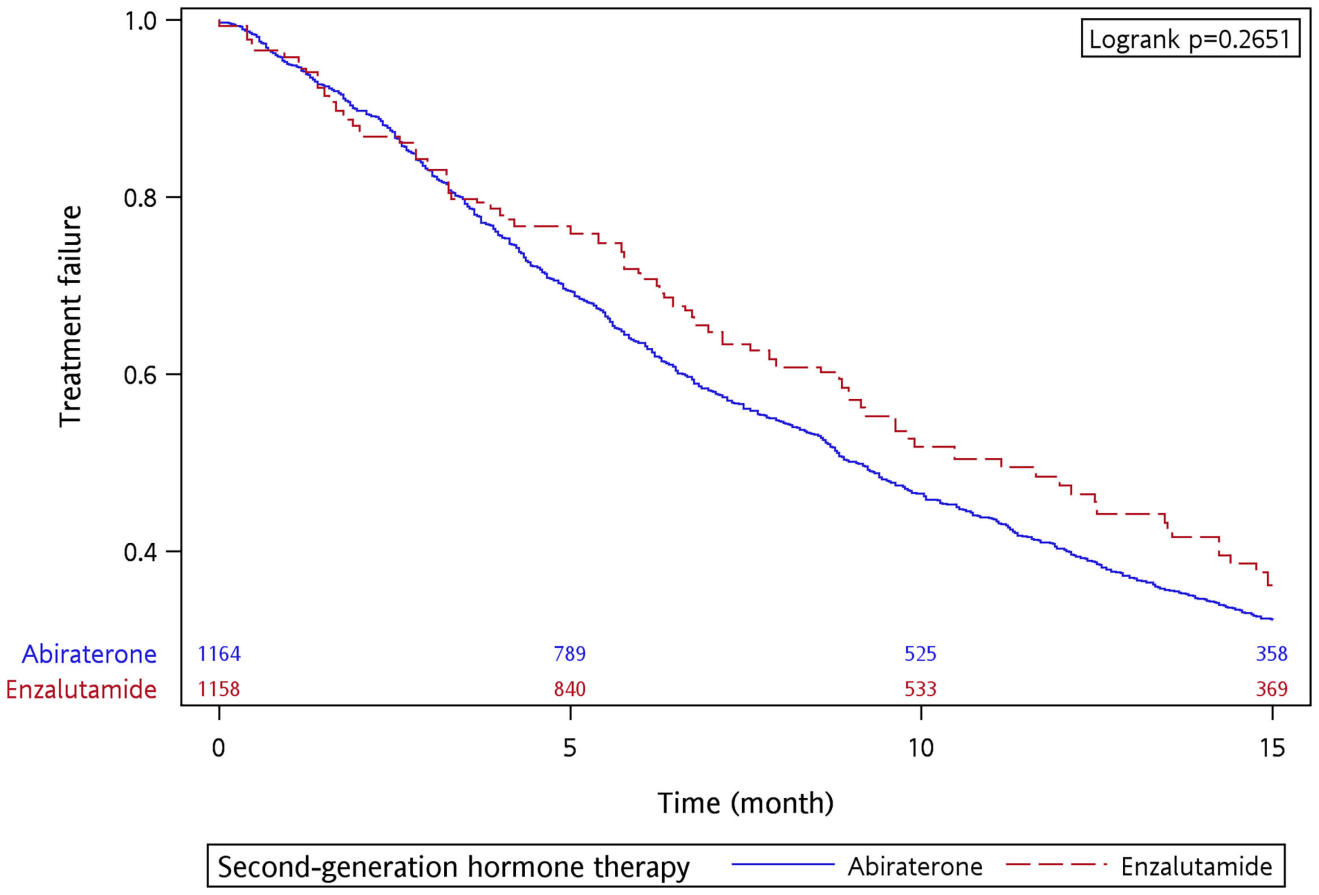
Time to treatment failure of abiraterone and enzalutamide in the 15-month follow-up period after applying IPTW.

There were 694 and 71 treatment failure events in the abiraterone and enzalutamide groups, respectively. The 15-month treatment failure rate was 67.81% and 65.21% for the abiraterone and enzalutamide groups, respectively. The median TTF was 9.07 and 9.90 months for the abiraterone and enzalutamide groups, respectively, with no significant difference between the two groups (*p* = 0.4416 by log-rank test) (**Supplementary Figure S3**). After the IPTW adjustment, the 15-month treatment failure rate was 67.79% and 63.84% for the abiraterone and enzalutamide groups, respectively. The median TTF was 9.07 and 11.13 months for the abiraterone and enzalutamide groups, respectively (**Figure 2**). A similar treatment effect was observed between the abiraterone and enzalutamide groups (*p* = 0.2651 by log-rank test). **Table 3** shows the results of the univariate and multivariable analyses after performing the IPTW. In the univariate analysis, the enzalutamide group had a lower risk of treatment failure than the abiraterone group (crude HR, 0.863, 95% CI 0.778–0.956, *p* = 0.005). However, the results of the multivariable analysis showed no significant intergroup difference (adjusted HR, 0.902, 95% CI 0.812–1.002, *p* = 0.0551). The multivariate analysis indicated that metastasis site, ADT duration (adjusted HR, 0.557, 95% CI 0.452–0.688, *p* < 0.0001), and diabetes mellitus (adjusted HR, 1.230, 95% CI 1.091–1.386, *p* = 0.0007) were factors associated with treatment failure.

**Table 3:** Cox proportional hazards model for time to treatment failure after IPTW.

	*N*	Number of events (%)	Univariate analysis			Multivariate analysis		
			Crude HR	95% CI	*p*-value	Adjusted HR	95% CI	*p*-value
Group								
Abiraterone	1,164	771 (66.24)						
Enzalutamide	1,158	678 (58.55)	0.863	0.778–0.956	0.005	0.902	0.812–1.002	0.0551
Age (years)								
45–64	467	282 (60.43)						
65–84	1,655	1,072 (64.74)	1.043	0.909–1.196	0.551	1.007	0.876–1.158	0.9195
≥85	199	101 (50.53)	0.724	0.579–0.907	0.0049	0.813	0.645–1.024	0.0790
Metastasis site								
No bone/visceral	1,027	596 (58.06)						
Bone metastasis	1,018	675 (66.25)	1.398	1.252–1.561	<0.0001	1.160	0.948–1.420	0.1504
Visceral metastasis	83	54 (65.74)	1.713	1.297–2.262	0.0002	1.496	1.082–2.069	0.0148
Bone + visceral	194	130 (66.77)	1.929	1.594–2.334	<0.0001	1.536	1.185–1.991	0.0012
Previous ADT								
No	6	4 (66.67)						
Yes	2,316	1,451 (62.65)	0.957	0.359–2.551	0.9293	1.821	0.668–4.969	0.2417
Docetaxel cycles								
0	53	33 (62.15)						
1–7	849	511 (60.14)	0.799	0.562–1.134	0.2089	1.217	0.828–1.788	0.3177
≥8	1,420	911 (64.18)	0.856	0.606–1.21	0.3793	1.315	0.899–1.923	0.1581
ADT duration								
≤12 months	157	117 (74.85)						
>12 months	2,165	1,338 (61.77)	0.550	0.455–0.664	<0.0001	0.557	0.452–0.688	<0.0001
CCI score								
≤7	850	485 (57.01)						
>7	1,472	970 (65.91)	1.463	1.311–1.632	<0.0001	1.211	0.984–1.49	0.0704
Comorbidity								
Hypertension	1,221	756 (61.91)	0.992	0.895–1.099	0.8729			
Dyslipidemia	595	367 (61.65)	0.945	0.84–1.064	0.3511			
Diabetes mellitus	545	377 (69.08)	1.261	1.122–1.418	0.0001	1.230	1.091–1.386	0.0007
Liver disease	199	125 (62.83)	1.162	0.968–1.396	0.1074			
Stroke	69	43 (62.5)	0.982	0.726–1.328	0.9038			
Coronary artery disease	328	201 (61.24)	1.041	0.897–1.208	0.597			
CHF	100	72 (72)	1.685	1.328–2.137	<0.0001	1.066	0.914–1.243	0.4172
CKD	193	97 (50.27)	0.924	0.752–1.136	0.4538			
COPD	165	103 (62.58)	0.976	0.799–1.193	0.8159			

HR, hazard ratio; CI, confidence interval; ADT, androgen deprivation therapy; CCI, Charlson Comorbidity Index.

### Sensitivity Analyses

#### Follow-Up of 24 Months

The median OS was 14.73 and 17.73 months for the abiraterone and enzalutamide groups, respectively (**Supplementary Figure S4**). The survival time was significantly longer for the enzalutamide group (*p* = 0.0295 by log-rank test), with a 24-month survival rate of 31.22% and 47.23% for the abiraterone and enzalutamide groups, respectively. After applying the IPTW, the median OS was 14.73 and 18.80 months for the abiraterone and enzalutamide groups, respectively (**Supplementary Figure S5**). The enzalutamide group had a significantly longer survival time than the abiraterone group with a 24-month follow-up (*p* = 0.0169 by log-rank test). The 24-month survival rate was 31.15% and 47.71% for the abiraterone and enzalutamide groups, respectively.

## Discussion

This study evaluated the comparative effectiveness of abiraterone and enzalutamide in patients with mCRPC by analyzing data obtained from the NHIRD in Taiwan. From the OS results, enzalutamide was associated with better OS for mCRPC than abiraterone in the Taiwan population. However, the TTF outcome revealed that there was no significant difference between the effectiveness of these two agents when treating mCRPC.

From the study’s main outcome, there were several mechanisms that might explain the superior effectiveness of enzalutamide over abiraterone. First, abiraterone is a CYP17 inhibitor that can block androgen synthesis by inhibiting the enzymes required for androgen biosynthesis. Unlike abiraterone, enzalutamide is a potent competitor for androgen binding to AR and can inhibit multiple steps in the AR signaling pathway, including nuclear translocation, androgen-mediated DNA binding, and coactivator recruitment (18). Second, a number of studies have shown that serum testosterone levels and the testosterone “bounce” phenomenon can predict the response to enzalutamide and abiraterone in CRPC. Although current evidence shows that enzalutamide has a higher rate of testosterone bounce than abiraterone, it does not show any difference in PSA progression-free survival (PFS) or OS between enzalutamide and abiraterone (19). Lastly, mutations within the AR ligand-binding domain in the prostate cancer cell line LNCaP constitute a common mechanism by which androgen withdrawal experiences resistance. A review of second-generation antiandrogens and studies (20) showed that clinically relevant mutations of AR (L702H, H875Y, T878A, T877A, F877L, and W741C) are resistant to flutamide and bicalutamide and that enzalutamide is still sensitive to mCRPC cells with T877A and W741C mutations. However, abiraterone resistance is derived from the intratumoral generation of androgens and CYP17A1 production in conjunction with a progesterone-responsive mutant AR (T877A). Thus, switching from abiraterone to an AR blocker might be beneficial (21). From our study results, enzalutamide was superior to abiraterone in terms of survival, which might be due to enzalutamide having better outcomes in the AR mutation (T877A) than abiraterone in mCRPC.

The OS rate in the current study showed no significant difference between the two groups, similar to the trends found in other Taiwan studies (13, 22). However, a retrospective study conducted using the NHIRD showed that abiraterone (53.7%) had a significantly higher overall mortality rate than enzalutamide (40.55%) (23). Otherwise, the current study’s results on survival time were more similar to those observed in clinical trials and cohort studies from other countries. In the COU-AA-301 and AFFIRM trials, the median survival time for patients with mCRPC in the post-chemotherapy setting was 14.8 and 18.4 months, respectively (4, 5). Norris et al. conducted a study in the UK that showed no significant difference in OS rates (15.3 vs. 22.2 months for abiraterone and enzalutamide, respectively) (24). A study conducted by Chowdhury et al. also reported no significant difference between abiraterone and enzalutamide in survival outcomes (10). However, the inconsistent results for OR rates (by log-rank test) and HR might be due to the fact that the HR changed over time in the early study period. Therefore, the enzalutamide group had significantly longer OS than the abiraterone group in the 24-month follow-up.

In the COU-AA-301 and AFFIRM trials, the median time to PSA progression of the patients with mCRPC in a post-chemotherapy setting was 10.2 and 8.3 months (4, 5), respectively. However, the TTF was longer in the current study than the time to PSA progression in the AFFIRM trial. The results of two studies indirectly comparing the two drugs showed that enzalutamide was superior to abiraterone in radiographic PFS, time to PSA progression, and PSA response rate (8, 25). A meta-analysis consisting of cohort studies showed that the PSA response rate was significantly greater in the enzalutamide group than in the abiraterone group (9). However, other studies whose results differed from those of the current study had differing populations (pre- and post-chemotherapy), TTF definition, and different regulations of insurance payments. A number of studies did, however, show a TTF outcome trend similar to our results. A European study using PCR data showed no significant difference between abiraterone and enzalutamide in time to progression (9.6 vs. 10.3 months for abiraterone and enzalutamide, respectively) (10). The other study in Taiwan also found no significant difference in PFS (7.3 vs. 9.5 months for abiraterone and enzalutamide, respectively) (13).

The multivariate analysis showed that older age, metastasis site, and ADT duration were the risk factors related to the outcome. Liu et al. also reported that age was a significant factor for OS (22). The results of the current study showing that metastatic site was a risk factor for OS were consistent with a study conducted by Gandalia et al., who reported that patients with bone plus visceral metastases had a higher mortality risk, followed by those with visceral metastasis and bone metastasis alone (26). Patients with a longer ADT duration had a lower risk of treatment failure in the study. Chang et al. and Di Stefano et al. also reported that patients with longer hormone-sensitive periods had better outcomes (13, 27). Furthermore, diabetes mellitus was another factor potentially related to survival outcome. Abdel-Rahman conducted a study that pooled data from three clinical trials, indicating that diabetes mellitus did not have a significant effect on the outcomes of chemotherapy-naive CRPC (28). However, other studies have shown that baseline metabolic syndrome and uncontrolled diabetes were significant risk factors related to poorer survival (29, 30). A retrospective study showed that there was no prognostic impact of dyslipidemia on ADT outcomes (31). However, a higher proportion of the enzalutamide group had dyslipidemia, given that frequent laboratory abnormalities such as hypertriglyceridemia and hypercholesterolemia were observed in the abiraterone group. This consequently gave the appearance of a lower mortality risk for the patients with dyslipidemia.

### Strengths and Limitations

To our knowledge, this is the first population-based study comparing the effectiveness of the two drugs for treating mCRPC in Taiwan. The use of the NHIRD covering over 99% of the Taiwan population allowed the present study to include almost every patient treated with abiraterone and enzalutamide in the country.

However, there are a number of limitations to this study. First, TTF was used as a surrogate outcome for time to PSA progression and radiographic PFS (32, 33) due to the lack of laboratory data and imaging data in the NHI database. However, assuming that all of the disease management steps followed the NHI program’s regulations, the administration of the two study drugs would not be allowed if the submitted documents showed disease progression, which could be representative of disease progression to a certain extent. Furthermore, other medical behaviors related to disease progression were also considered in the study outcome, such as the addition of new treatment for prostate cancer or diagnosis of new metastases. Second, the study did not consider important prognostic factors such as the Gleason score, metastatic volume, testosterone level, PSA level at nadir, and the Eastern Cooperative Oncology Group performance status (13). Third, the number of patients in each group differed significantly, due to the difference in time between the introduction of the two drugs. Therefore, the study applied IPTW to produce a pseudo-population to assess the comparative effectiveness. However, IPTW may cause extreme weights which would increase bias effect (omission of interaction effects or misspecification of functional forms of covariates) and enhance treatment effect (17, 34). Lastly, some of the medical management of the self-paid items and the participation in clinical trials could not be obtained from the NHIRD. For example, records of certain therapeutic agents for mCRPC such as cabazitaxel and radium-223 were unavailable in the database. In fact, abiraterone and enzalutamide had been introduced in Taiwan in 2013 and 2015, respectively. As a result, the prescription records prior to this time could not be obtained until they were covered by the NHI system. The usage time might therefore be underestimated or overestimated.

## Conclusion

In conclusion, enzalutamide was associated with better OS for mCRPC than abiraterone in the Taiwan population. Our study revealed that there was no statistically significant difference in TTF between enzalutamide and abiraterone. Metastasis site and ADT duration were risk factors related to treatment failure and mortality in patients with mCRPC treated with second-generation hormone therapy.

## Data Availability Statement

The data analyzed in this study are subject to the following licenses/restrictions: Data are available from the National Health Insurance Research Database (NHIRD) published by the Bureau of National Health Insurance (BNHI) of the Ministry of Health and Welfare. Requests to access these datasets should be directed to C-YC (jk2975525@hotmail.com).

## Author Contributions

All authors listed have made a substantial, direct, and intellectual contribution to the work and approved it for publication.

## Funding

This work was supported by grants from the Kaohsiung Medical University Research Foundation (KMU-M1100018), Kaohsiung Medical University (KMU-S109032), and Kaohsiung Medical University Hospital (KMUH108-M819).

## Author Disclaimer

The conclusions presented in this study are those of the authors and do not necessarily reflect the views of the BNHI, Ministry of Health and Welfare.

## Conflict of Interest

The authors declare that the research was conducted in the absence of any commercial or financial relationships that could be construed as a potential conflict of interest.

## Publisher’s Note

All claims expressed in this article are solely those of the authors and do not necessarily represent those of their affiliated organizations, or those of the publisher, the editors and the reviewers. Any product that may be evaluated in this article, or claim that may be made by its manufacturer, is not guaranteed or endorsed by the publisher.
