# Supplementary material for: Comparative Effectiveness of Abiraterone and Enzalutamide in Patients With Metastatic Castration-Resistant Prostate Cancer in Taiwan
Source: Front Oncol. 2022 Mar 7;12:822375. doi: 10.3389/fonc.2022.822375 (PMC8940330; doi:10.3389/fonc.2022.822375)
Supplement: Supplementary file 1 [file DataSheet_1.pdf]

Supplementary Material

Comparative Effectiveness of Abiraterone and Enzalutamide in Patients with Metastatic Castration-Resistant Prostate Cancer in Taiwan

Table S1. Baseline characteristics after applying IPTW
Table S2. ICD code for diseases

Table S1. Baseline characteristics after applying IPTW

	N	%	Abiraterone		Enzalutamide		SMD
			N	%	N	%	
**Total**	2322		1164		1158		
**Age** (years)							
Mean (±SD)	72.36	±12.56	72.83	±9.2	71.87	±28.43	0.05
45-64	467	20.12	234	20.11	233	20.14	0
65-84	1655	71.29	837	71.89	819	70.70	0.03
≥ 85	199	8.58	93	8.00	106	9.17	0.04
**Metastasis site**							
No bone/visceral	1027	44.23	504	43.33	523	45.14	0.04
Bone metastasis	1018	43.85	512	43.98	506	43.73	0
Visceral metastasis	83	3.56	41	3.54	41	3.57	0
Bone + Visceral	194	8.36	107	9.16	88	7.56	0.06
**Previous treatment**							
None	5	0.22	5	0.43	0	0	-
RP/RT only	1	0.04	1	0.09	0	0	-
Hormone only	1763	75.94	843	72.44	920	79.46	0.16
Hormone + RP/RT	553	23.80	315	27.04	238	20.54	0.15
**Docetaxel cycles**							
0	53	2.30	26	2.26	27	2.35	0.01
1-7	849	36.56	424	36.41	425	36.71	0.01
≥ 8	1420	61.14	714	61.33	706	60.94	0.01
**ADT duration**							
≤ 12 mon	157	6.75	73	6.29	83	7.21	0.04
> 12 mon	2165	93.25	1091	93.71	1074	92.79	
**CCI score**							
≤ 7	850	36.60	416	35.72	434	37.49	0.04
> 7	1472	63.40	748	64.28	724	62.51	

Table S1. Baseline characteristics after applying IPTW (continued)

	N	%	Abiraterone		Enzalutamide		SMD
			N	%	N	%	
**Comorbidity**							
Hypertension	1221	52.59	604	51.91	617	53.26	0.03
Dyslipidemia	595	25.64	224	19.20	372	32.12	0.30
Diabetes mellitus	545	23.49	273	23.49	272	23.49	0
Liver disease	199	8.59	99	8.50	101	8.69	0.01
Stroke	69	2.99	69	5.96	0	0.00	-
Coronary artery disease	328	14.11	171	14.73	156	13.50	0.04
Congestive heart failure	100	4.29	51	4.39	48	4.18	0.01
Chronic kidney disease	193	8.33	108	9.27	86	7.39	0.07
COPD	165	7.09	96	8.25	69	5.92	0.09

SMD: standard mean difference; SD: standard deviation; RP: radical prostatectomy; RT: radiation therapy; ADT: androgen deprivation therapy; CCI: Charlson Comorbidity Index; COPD: chronic obstructive pulmonary disease

Table S2. ICD code for diseases

Disease	ICD-9-CM	ICD-10-CM
**Comorbidities**		
**Hypertension**	401-405	I10, I11, I12, I13, N26.2
**Dyslipidemia**	272	E71.30, E75.2, E75.3, E75.5, E75.6, E77.0, E77.1, E77.8, E77.9, E78.0-78.6, E78.70, E78.8, E78.9, E79.1, E79.2, E79.8, E79.9, E88.1, E88.2, E88.89
**Diabetes mellitus**	250	E10.0- E14.9
**Liver disease**	571.2, 571.4-571.6, 572.3-572.8, 456.0-456.2	B18, K70.0-K70.3, K70.9, K71.6-K71.5, K71.7, K73, K74, K76.0, K76.2-K76.4, K76.8, K76.9, Z94.4, I85.0, I85.9, I86.4, I98.2, K70., K71.1, K72.1, K72.9, K76.5-K76.7
**Stroke**	430-438	I60-I63, I65-I69, G45
**Cardiac artery disease**	410-414	I20, I21, I22, I24, I25
**Congestive heart failure**	428	I50
**Chronic kidney disease**	585	N18.4, N18.5, N18.6, N18.9
**COPD**	491, 492, 496	J41-J44
**Obesity**	278.0	E66
**Secondary malignant neoplasm**		
**Lymph node**	196.0-196.9	C77.0-C77.9
**Bone**	198.5	C79.51, C79.52
**Liver**	197.7	C78.7
**Lung**	197.0	C78.00, C78.01, C78.02
**CNS**	198.3, 198.4	C79.31, C79.32, C79.40, C79.49
**Adrenal gland**	198.7	C79.70, C79.71, C79.72
**Peritoneum**	197.6	C78.6
