# Supplementary material for: Comparative Effectiveness of Abiraterone and Enzalutamide in Patients With Metastatic Castration-Resistant Prostate Cancer in Taiwan
Source: Front Oncol. 2022 Mar 7;12:822375. doi: 10.3389/fonc.2022.822375 (PMC8940330; doi:10.3389/fonc.2022.822375)
Supplement: Supplementary file 2 [file DataSheet_2.pdf]

Supplementary Material

Comparative Effectiveness of Abiraterone and Enzalutamide in Patients with Metastatic Castration-Resistant Prostate Cancer in Taiwan

Figure S1. Results of the study population selection
Figure S2. Overall survival of crude analysis
Figure S3. Time to treatment failure of crude analysis
Figure S4. Overall survival of crude analysis with 24-month follow-up period
Figure S5. Overall survival after performing IPTW with 24-month follow-up period

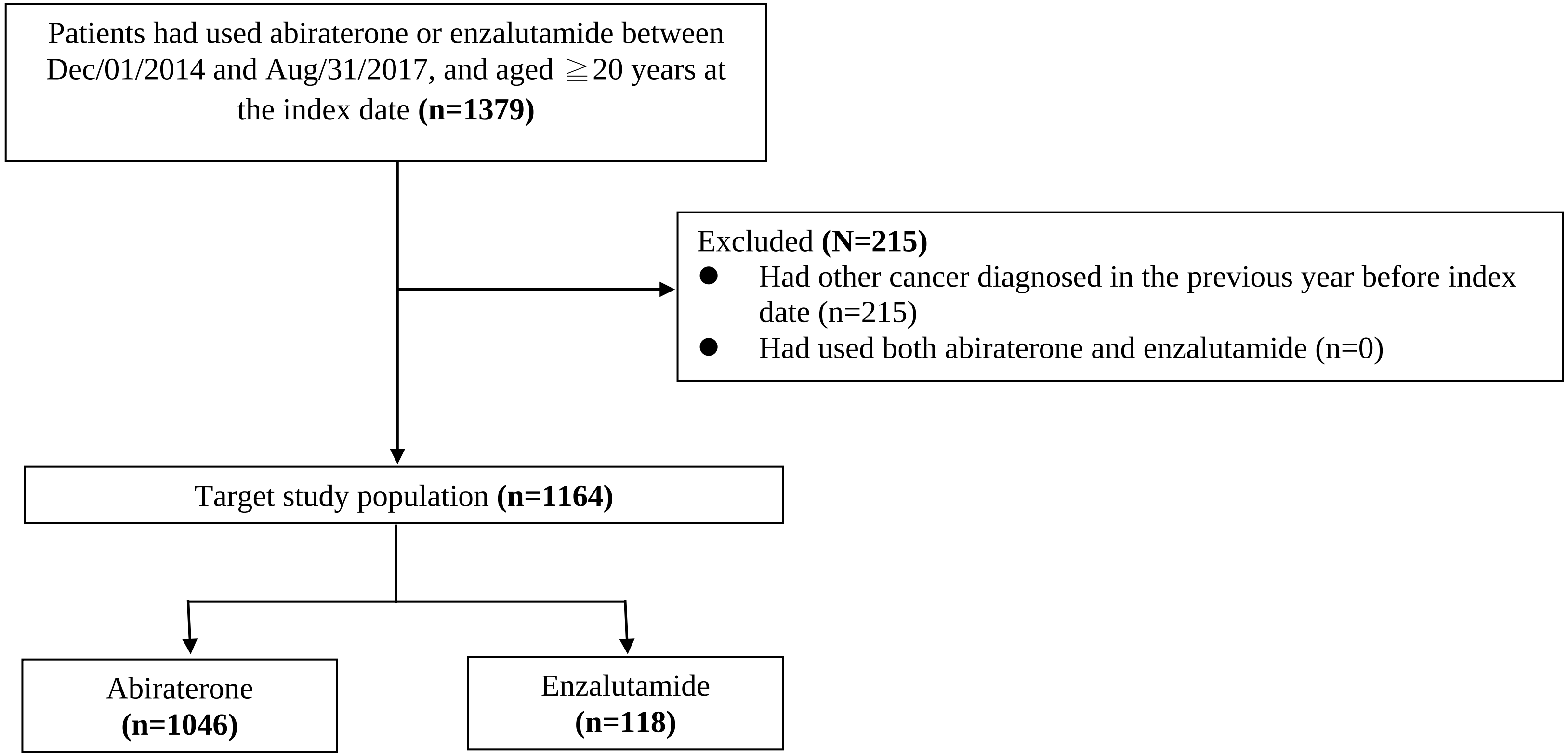

Figure S1. Results of the study population selection

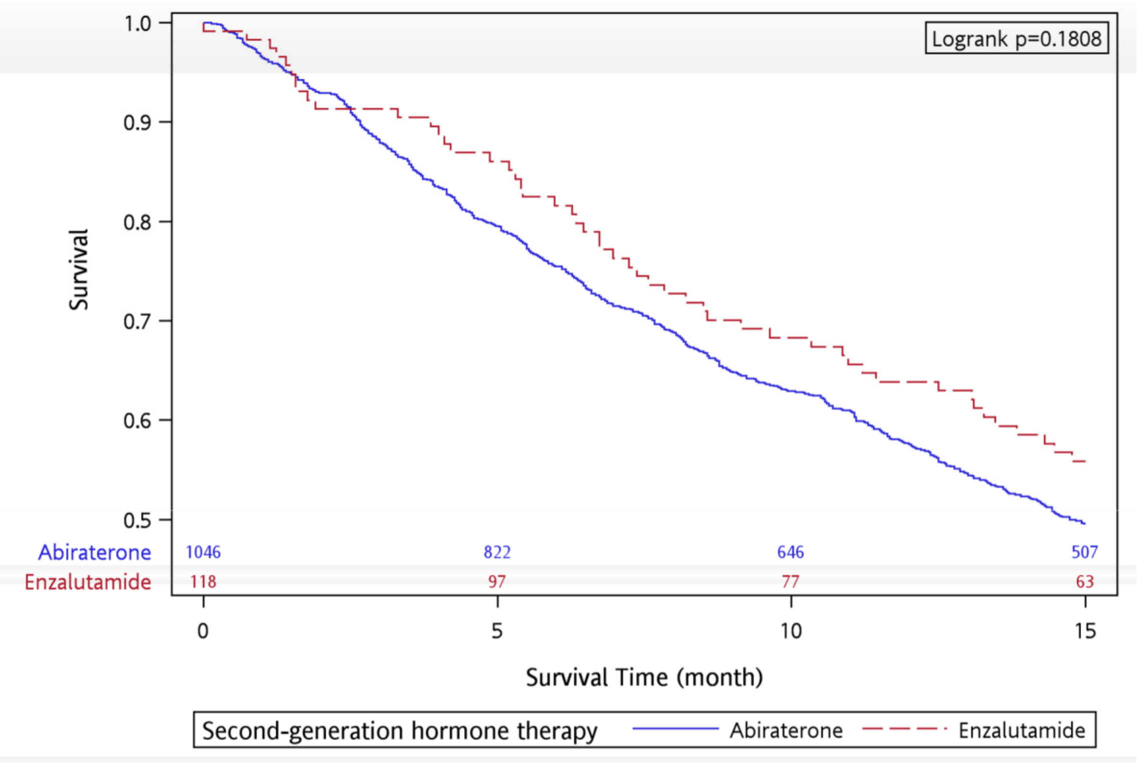

Figure S2. Overall survival of crude analysis

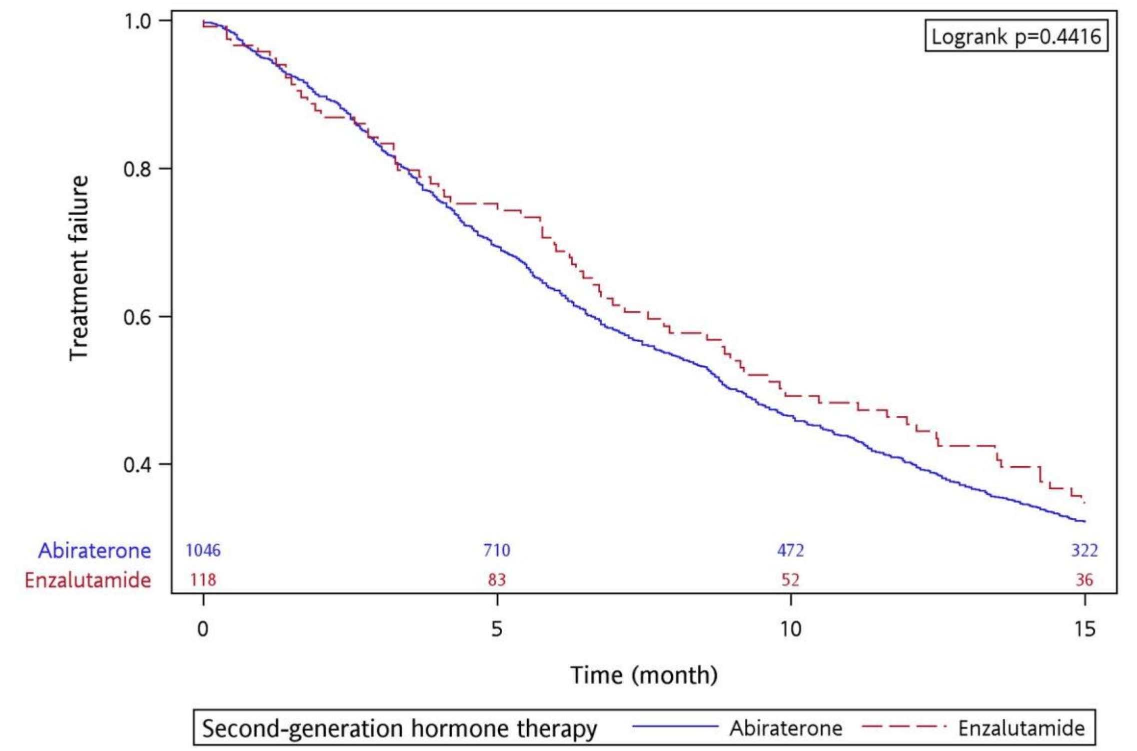

Figure S3. Time to treatment failure of crude analysis

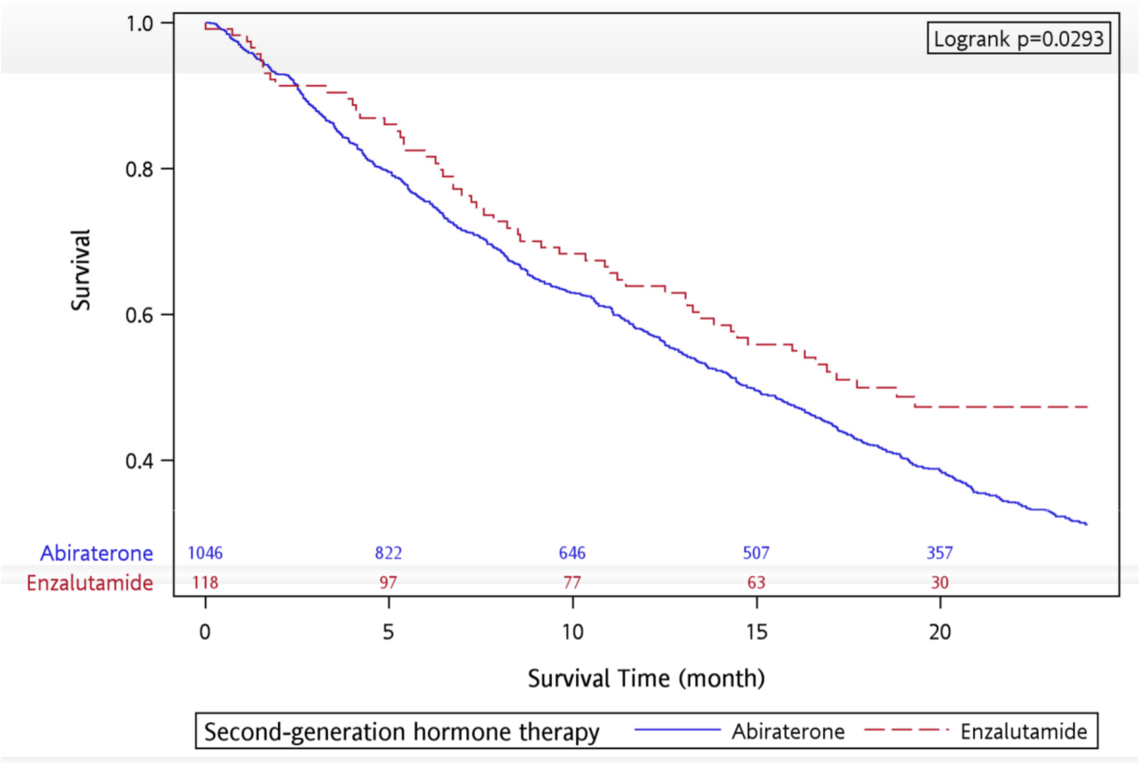

Figure S4. Overall survival of crude analysis with 24-month follow-up period

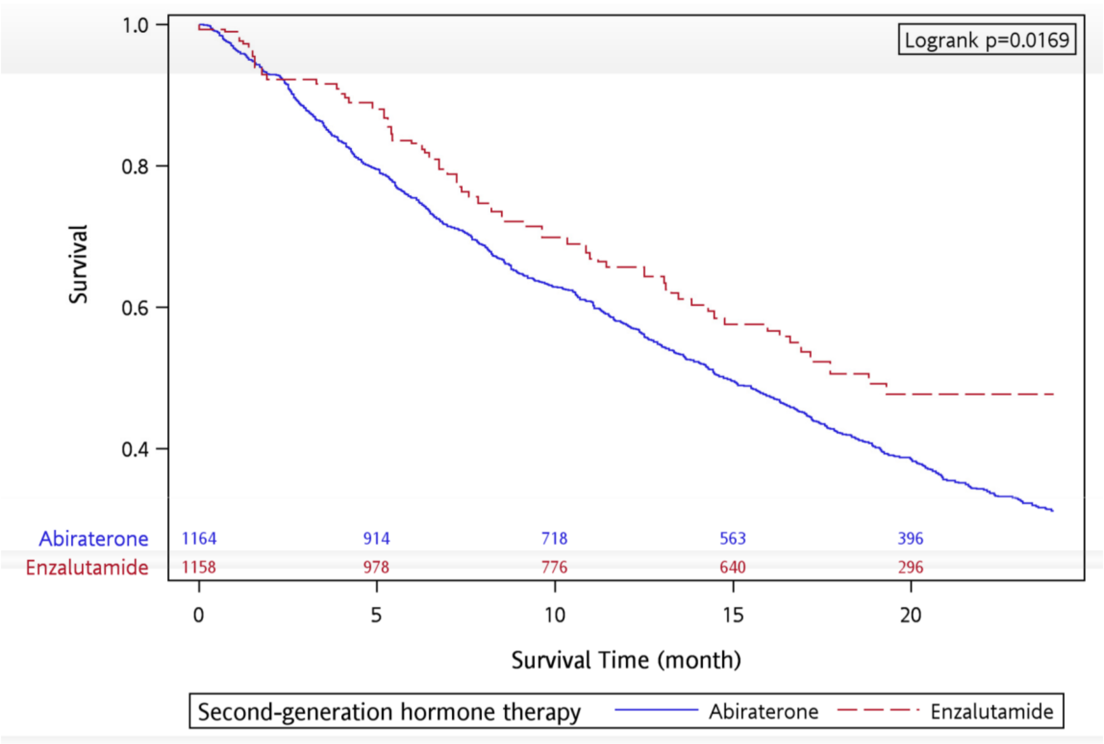

Figure S5. Overall survival after performing IPTW with 24-month follow-up period
